# Assembly of genomic reads of elite indica rice cultivar onto 2101 reference bacterial genomes for identification of co-sequenced endophytic bacteria

**DOI:** 10.1016/j.dib.2017.03.036

**Published:** 2017-03-31

**Authors:** Latha Battu, Mettu Madhavi Reddy, Burragoni Sravanthi Goud, Kayalvili Ulaganathan, Kandasamy Ulaganathan

**Affiliations:** Centre for Plant Molecular Biology, Osmania University, Hyderabad 500007, Telangana, India

## Abstract

Reference based assembly of genomic reads of the elite indica rice cultivar RP Bio-226 was carried out against 2101 reference bacterial genomes using Bowtie-2 genome assembly tool. Five types of data: Number of paired end reads concordantly aligned exactly only once, number of paired end reads concordantly aligned more than once, number of mates that make the pairs aligned exactly only once, number of mates that make the pairs aligned more than once and overall percentage of alignment were collected. Interpretation of the results and identification of endophytes based on these alignment statistics are described in detail in our research article “L.Battu, M.M. Reddy, B.S.Goud, K.Ulaganathan, K.Ulaganathan, Genome inside genome:NGS based identification and assembly of endophytic *Sphingopyxis granuli* and *Pseudomonas aeruginosa* genomes from rice genomic reads, Genomics (in press)”.

**Specifications Table**

**Table:** 

Subject area	Biology
More specific subject area	Genomics
Type of data	Genome assembly data
How data was acquired	Computational reference based genome assembly
Data format	Analyzed
Experimental factors	Adapters were removed from the genomic reads
Experimental features	The pre-processing of raw reads was done with FastQC and the adapters were removed with Cutadapt tool. After pre-processing, the reads were aligned to the reference genome by using Bowtie2 (ver. 2.2.4). The reference genomes of 2101 bacterial species including *Pseudomonas aeruginosa* PAO1 and *Sphingopyxis granuli* were downloaded from NCBI. Reference based assembly of the reads against these reference genomes involved, indexing of the reference genomes and alignment of reads to the reference and creation of SAM files.
Data source location	Not applicable
Data accessibility	Data is with this article

**Value of the data**

• The data listed in tabular form provides information on assembly of reads of *Oryza sativa* indica cultivar RP Bio-226 onto 2101 reference bacterial genomes.
• This data can be compared to similar alignment of genomic reads from other rice cultivars onto reference bacterial genomes.
• The alignment data explains a new method of identification of endophytes from plant genomic reads and can be followed in other plants.

## 1 Data

Results of reference based assembly of genomic reads of elite indica rice cultivar RP Bio-226 in tabular form are the data mentioned in this paper. The tabulated data include five types of alignment statistics data: Number of paired end reads concordantly aligned exactly only once, number of paired end reads concordantly aligned more than once, number of mates that make the pairs aligned exactly only once, number of mates that make the pairs aligned more than once and overall percentage of alignment. (Supplementary Table-1) Additionally, genomes which showed maximum alignment with rice reads with respect to the above 5 different types of data are tabulated separately and enclosed (Table 1, Table 2, Table 3, Table 4, Table 5).

## 2 Experimental design, materials and methods

Total DNA was isolated from the Leaves of in vitro grown *Oryza sativa* indica cultivar RP Bio-226 plants and sequencing library was prepared [1]. Whole genome sequencing was carried out with Illumina_Nextseq. 500 system (Illumina, San Diego, CA). The raw data files in Fastq format were used for further analysis. The pre-processing of raw reads was done with FastQC and the adapters were removed with Cutadapt tool [2], [3]. After pre-processing, the reads were aligned to the reference genome by using Bowtie2 (ver. 2.2.4) [4]. The reference genomes of 2101 bacterial species including *Pseudomonas aeruginosa* PAO1 and *Sphingopyxis granuli* were downloaded from NCBI. Reference based assembly of the reads against these reference genomes involved, indexing of the reference genomes and alignment of reads to the reference and creation of SAM files. Samtools (ver 0.1.18) was used for further analysis [5]. SAM files were converted into binary BAM files, sorted and indexed by using the ‘view’, ‘sort’ and ‘index’ functions of SAMtools. The consensus sequences were created with Samtools. Genome annotation was carried out with RAST and BaySys servers [6], [7]. The tRNAs were identified with tRNAscan-SE software and rRNAs were identified with Rfam software [8], [9].

## Figures and Tables

**Table 1:** Reference based assembly of RP Bio-226 genomic reads on to bacterial genome: List of genomes to which more than 10,000 paired end reads aligned concordantly exactly only once.

**Table 2:** Reference based assembly of RP Bio-226 genomic reads on to bacterial genome: List of genomes to which more than 10,000 paired end reads aligned concordantly more than once.

S.No	Name of the Bacterial Genomes used as reference	Reads aligned concordantly exactly 1 time	Reads aligned concordantly >1 time	Mates that make up the pairs aligned concordantly or discordantly exactly 1 time	Mates that make up the pairs aligned concordantly or discordantly >1 time	Overall Alignment rate %
1	*Cylindrospermum stagnale*	2294	35317	3106	77525	0.22
2	*Geitlerinema sp.*	1	32249	3	84912	0.22
3	*Oscillatoria acuminata*	132	31314	180	83284	0.21
4	*Nostoc azollae*	54	31289	38	70590	0.19
5	*Microcystis panniformis*	0	31104	3	85117	0.21
6	*Microcystis aeruginosa*	16	31094	229	85131	0.21
7	*Cyanobacterium*	0	30463	8	82316	0.21
8	*Cyanobacterium endosymbiont*	0	30463	8	82316	0.21
9	*Nostoc punctiforme*	1	29246	1	88219	0.21
10	*Oscillatoriales cyanobacterium*	0	28541	3	81824	0.20
11	*Oscillatoria nigroviridis*	4	28176	5	96119	0.22
12	*Fischerella sp.*	431	28127	173	73901	0.19
13	*Acaryochloris marina*	10	27985	8	75714	0.19
14	*Nodularia spumigena*	4	27513	100	80485	0.20
15	*Synechococcus elongatus*	4	26252	29	67922	0.17
16	*Cyanobium gracile*	1	26016	25	70894	0.18
17	*Anabaena variabilis*	4	25773	0	80169	0.19
18	*Trichodesmium erythraeum*	6	25746	53	88351	0.20
19	*Arthrospira platensis*	0	25699	2	91181	0.21
20	*Crinalium epipsammum*	5	24247	17	88945	0.20
21	*Chroococcidiopsis thermalis*	1	20908	6	73861	0.17
22	*Microcoleus sp.*	4	20405	25	89544	0.19
23	*Dactylococcopsis salina*	52	20361	175	72443	0.16
24	*Candidatus Atelocyanobacterimthalassa*	0	19925	4	69551	0.16
25	*Pseudanabaena.sp.*	11	17760	48	74103	0.16
26	*Rivularia sp.*	0	17695	0	73210	0.16
27	*Halothece sp.*	209	17504	1301	62653	0.14
28	*Planktothrix agardhii*	268	15578	1982	70470	0.15
29	*Cyanobacterium aponinum*	0	15458	0	48759	0.11
30	*Chamaesiphon minutus*	1	14566	4	68114	0.14
31	*Cyanobacterium stanieri*	34	14415	630	49301	0.11
32	*Stanieria cyanosphaera*	2734	11866	8319	72035	0.16
33	*Geminocystis herdmanii*	2683	10717	36938	16238	0.12

**Table 3:** Reference based assembly of RP Bio-226 genomic reads on to bacterial genome: List of genomes to which more than 10,000 mates that make up the pairs aligned concordantly or discordantly exactly 1 time.

**Table 4:** Reference based assembly of RP Bio-226 genomic reads on to bacterial genome: List of genomes to which more than 10,000 mates that make up the pairs aligned concordantly or discordantly more than once.

S.No	Name of the Bacterial Genomes used as reference	Reads aligned concordantly exactly 1 time	Reads aligned concordantly >1 time	Mates that make up the pairs aligned concordantly or discordantly exactly 1 time	Mates that make up the pairs aligned concordantly or discordantly >1 time	Overall Alignment rate %
1	*Oscillatoria nigro-viridis*	4	28176	5	96119	0.22
2	*Arthrospira platensis*	0	25699	2	91181	0.21
3	*Microcoleus sp.*	4	20405	25	89544	0.19
4	*Crinalium epipsammum*	5	24247	17	88945	0.20
5	*Trichodesmium erythraeum*	6	25746	53	88351	0.20
6	*Nostoc punctiforme*	1	29246	1	88219	0.21
7	*Microcystis aeruginosa*	16	31094	229	85131	0.21
8	*Microcystis panniformis*	0	31104	3	85117	0.21
9	*Geitlerinema.sp.*	1	32249	3	84912	0.22
10	*Oscillatoria acuminata*	132	31314	180	83284	0.21
11	*Cyanobacterium*	0	30463	8	82316	0.21
12	*Cyanobacterium endosymbiont*	0	30463	8	82316	0.21
13	*Oscillatoriales cyanobacterium*	0	28541	3	81824	0.20
14	*Nodularia spumigena*	4	27513	100	80485	0.20
15	*Anabaena variabilis*	4	25773	0	80169	0.19
16	*Cylindrospermum stagnale*	2294	35317	3106	77525	0.22
17	*Acaryochloris marina*	10	27985	8	75714	0.19
18	*Pseudanabaena.sp.*	11	17760	48	74103	0.16
19	*Fischerella.sp.*	431	28127	173	73901	0.19
20	*Chroococcidiopsis thermalis*	1	20908	6	73861	0.17
21	*Rivularia sp.*	0	17695	0	73210	0.16
22	*Dactylococcopsis salina*	52	20361	175	72443	0.16
23	*Stanieria cyanosphaera*	2734	11866	8319	72035	0.16
24	*Cyanobium gracile*	1	26016	25	70894	0.18
25	*Nostoc azollae*	54	31289	38	70590	0.19
26	*Planktothrix agardhii*	268	15578	1982	70470	0.15
27	*Candidatus Atelocyanobacterim thalassa*	0	19925	4	69551	0.16
28	*Chamaesiphon minutus*	1	14566	4	68114	0.14
29	*Synechococcus elongatus*	4	26252	29	67922	0.17
30	*Halothece sp.*	209	17504	1301	62653	0.14
31	*Cyanobacterium stanieri*	34	14415	630	49301	0.11
32	*Cyanobacterium aponinum*	0	15458	0	48759	0.11

**Table 5:** Reference based assembly of RP Bio-226 genomic reads on to bacterial genomes: List of genomes to which at least 0.1% paired end reads are aligned concordantly or discordantly.

## Footnotes

Appendix A Supplementary data associated with this article can be found in the online version at 10.1016/j.dib.2017.03.036.
